# Supplementary material for: Rif1 S-acylation mediates DNA double-strand break repair at the inner nuclear membrane
Source: Nat Commun. 2019 Jun 10;10:2535. doi: 10.1038/s41467-019-10349-z (PMC6557901; doi:10.1038/s41467-019-10349-z)
Supplement: Supplementary file 1 — Supplementary Information [file 41467_2019_10349_MOESM1_ESM.pdf]

Supplementary Information for:

Rif1 *S*-acylation mediates DNA double-strand break repair at the inner nuclear membrane

Fontana et al.

Supplementary Figures

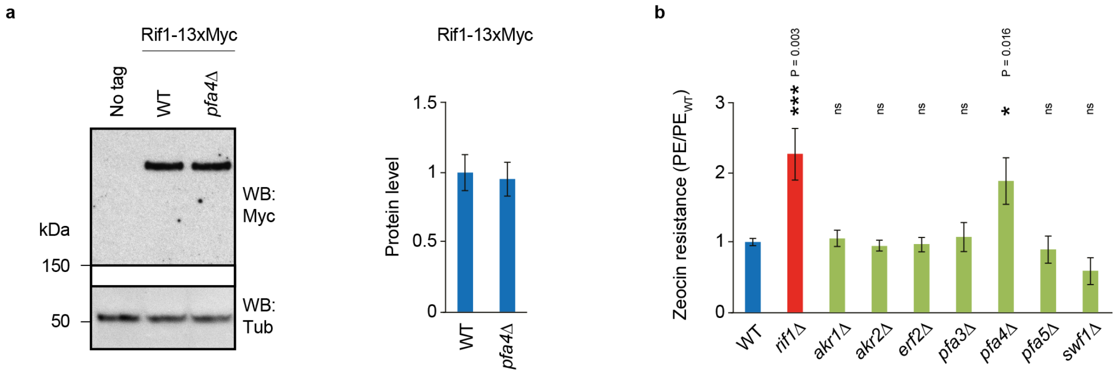

Supplementary Figure 1: Rif1 is not destabilized in absence of Pfa4. (a) Western blot analysis and quantitation of Myc-tagged Rif1 in lysates of wild-type and *pfa4*Δ cells. Protein levels relative to wild-type are presented as mean values ± s.e.m. (n = 3 independent experiments). (**b**) Cell viability in the presence of Zeocin (70 µg/ml) of strains deleted for *RIF1* or for genes encoding the seven yeast DHHC palmitoyl transferases. Data are presented as mean values ± s.e.m. (n = 6 independent experiments). Statistical analysis by one-way Anova followed by a post-hoc Tukey–Kramer multiple comparison test, comparing wild-type to the indicated mutants.

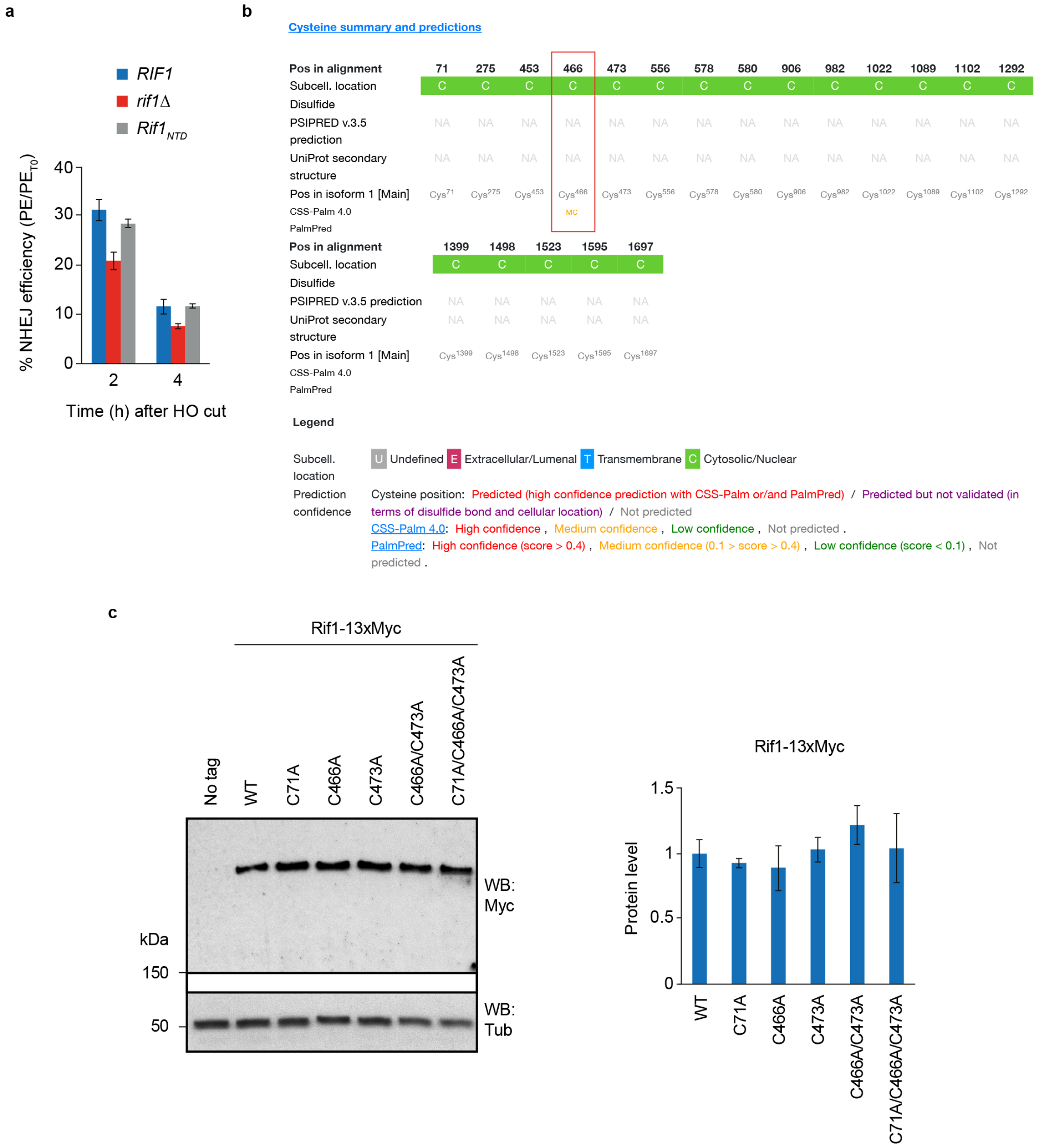

Supplementary Figure 2: Prediction of *S*-palmitoylation and stability of Rif1 cysteine cluster 2 mutants. (**a**) Rif1$_{NTD}$ is NHEJ-proficient. NHEJ efficiency for the indicated strains measured by cell viability following 2 or 4 h of HO-endonuclease induction. Data are presented as mean values ± s.e.m. (n = 3 independent experiments). (**b**) Swisspalm (CSS-Palm server) predicts *S. cerevisiae* Rif1 C466 as a potential site of *S*-palmitoylation. (**c**) Western blot analysis and quantitation of Myc-tagged Rif1 harboring the indicated amino acid substitutions. Protein levels relative to wild-type are presented as mean values ± s.e.m. (n = 3 independent experiments).

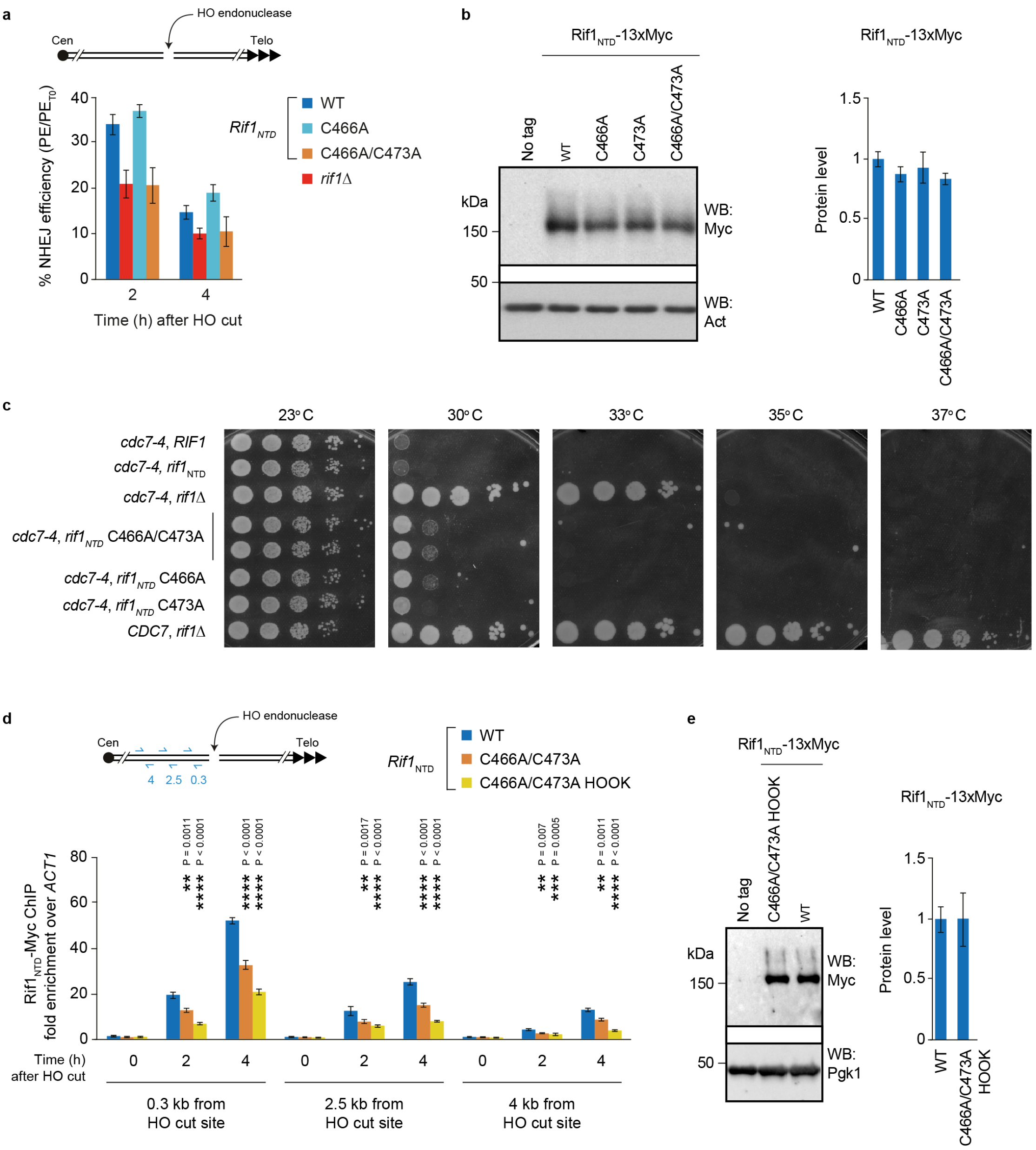

Supplementary Figure 3: Functional analyses of Rif1$_{NTD}$ and mutants. (**a**) NHEJ efficiency of strains deleted for *RIF1* or harboring Rif1$_{NTD}$ with the indicated mutations. Cell viability was measured following 2 or 4 h of DSB-induction at the *MAT* locus. Data are presented as mean values ± s.e.m. (n = 3 independent experiments). (**b**) Assessment of Rif1$_{NTD}$ mutant protein stability. Western blot and quantitation of Myc-tagged Rif1$_{NTD}$ harboring the indicated amino acid substitutions. Protein levels relative to wild-type are presented as mean values ± s.e.m. (n = 3 independent experiments). (**c**) Replication assay. The indicated strains were grown overnight in YPAD at 25 °C. Serial 10-fold dilutions were spotted onto YPAD plates and incubated at the indicated temperatures for 48 h. As expected, a *RIF1* deletion rescues the temperature sensitivity of *cdc7-4* replication mutants, allowing the activation of origins that are normally suppressed by Rif1. In contrast, Rif1$_{NTD}$ C466A/C473A behaves similar to wild-type Rif1, indicating the mutant is proficient in suppressing replication origin firing. (**d**) Association

of wild-type and the indicated mutant versions of Rif1$_{NTD}$-Myc with an induced DSB at the *MAT* locus. Results obtained with the indicated primers are reported as fold enrichment relative to *ACT1* ± s.e.m. (n = 6 independent experiments). Statistical analysis by one-way Anova and a post-hoc Tukey–Kramer multiple comparison test, comparing wild-type to the indicated mutants. (**e**) Assessment of Rif1$_{NTD}$ mutant protein stability as described for panel **b**.

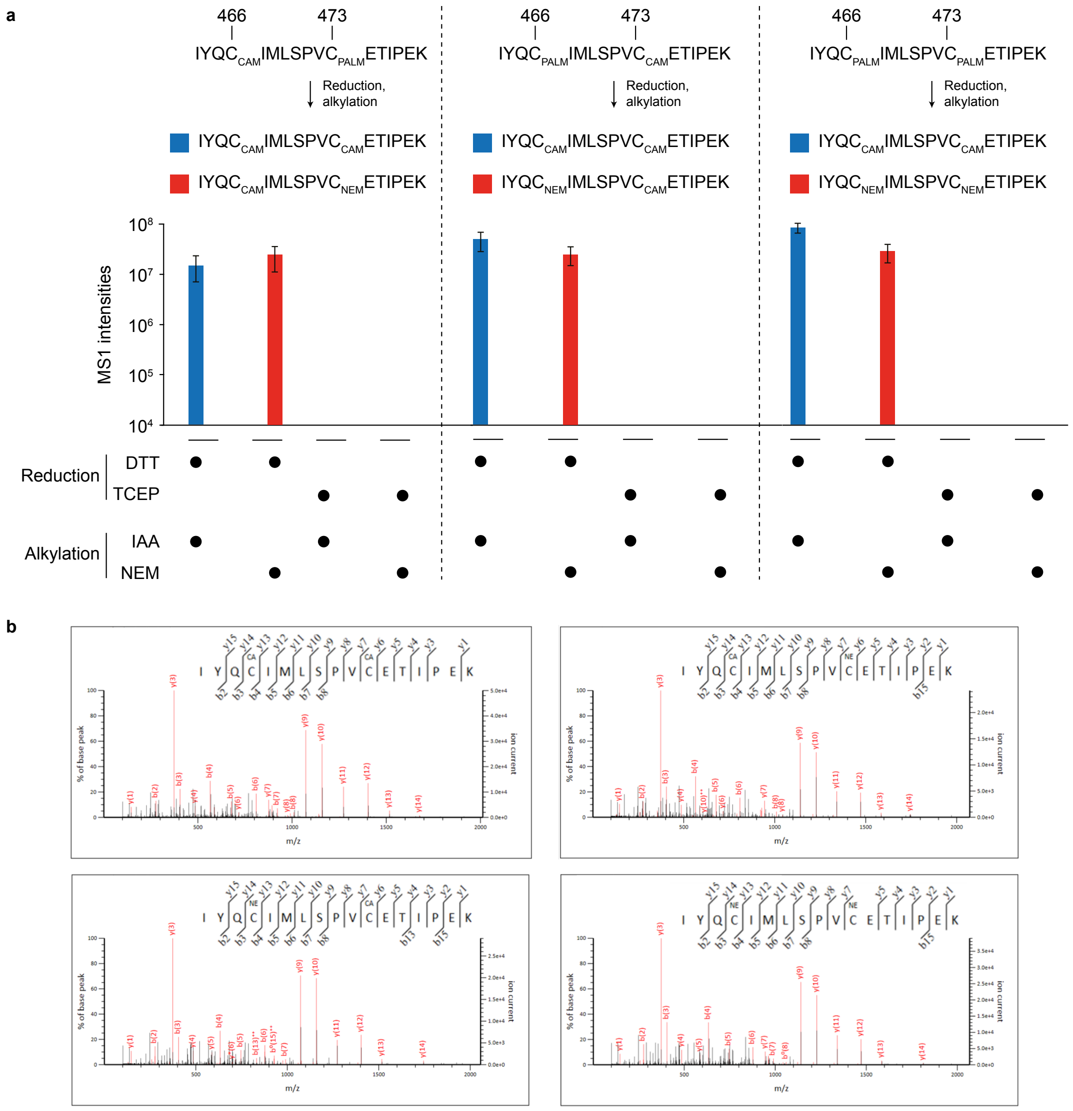

c

Precursors and fragments used for relative quantitative analyses in Skyline

peptide	m/z [2+]	y3	y9	y10	y11	y12	rt [min]
$C466_{CAM}$-$C473_{CAM}$	1041.5	373.2082	1072.5343	1159.5664	1272.6504	1403.6909	29.1
$C466_{CAM}$-$C473_{NEM}$	1075.1	373.2082	1140.5605	1227.5926	1340.6766	1471.7171	32.4
$C466_{NEM}$-$C473_{CAM}$	1075.1	373.2082	1072.5343	1159.5664	1272.6504	1403.6909	33.2
$C466_{NEM}$-$C473_{NEM}$	1109.1	373.2082	1140.5605	1227.5926	1340.6766	1471.7171	35.8

Supplementary Figure 4: Removal of Rif1 palmitoylation by DTT but not TCEP, followed by chemical labeling. (**a**) The sequence and cysteine modifications of the three synthetic peptides designed on the tryptic fragment of Rif1 spanning residues 463 to 479 and containing C466 or C473 is shown on the top (PALM denotes palmitoylation). The peptides were subjected to reduction with 40 mM DTT or TCEP for 2 h at 56 °C. As shown by mass spectrometry, DTT, but not TCEP, cleaved the cysteine-acyl thioester bond to remove the palmitoyl moieties from C466 and C473 allowing subsequent alkylation in the presence of 90 mM iodoacetamide (IAA) or N-ethylmaleimide (NEM) for 1h at 25 °C. Thus, TCEP can be

used to reduce disulfide bridges in biological protein samples with *S*-acylation being preserved. Mass spectrometry results are presented as mean signal values +/- s.e.m. (n = 3 independent experiments). MS1 intensities are shown in a logarithmic scale. (**b**) Four representative HCD spectra of peptides carrying CAM (CA) and/or NEM (NE)-labeled C466/C473 are showing a high similarity, both for the fragments detected as well as their relative intensities. The sequence, the cysteine modification, and the detected fragments are shown. (**c**) Table illustrating the precursors (m/z) of the peptides used for the PRM analysis, the fragments used for relative quantitation in Skyline and the retention time for each individual peptide.

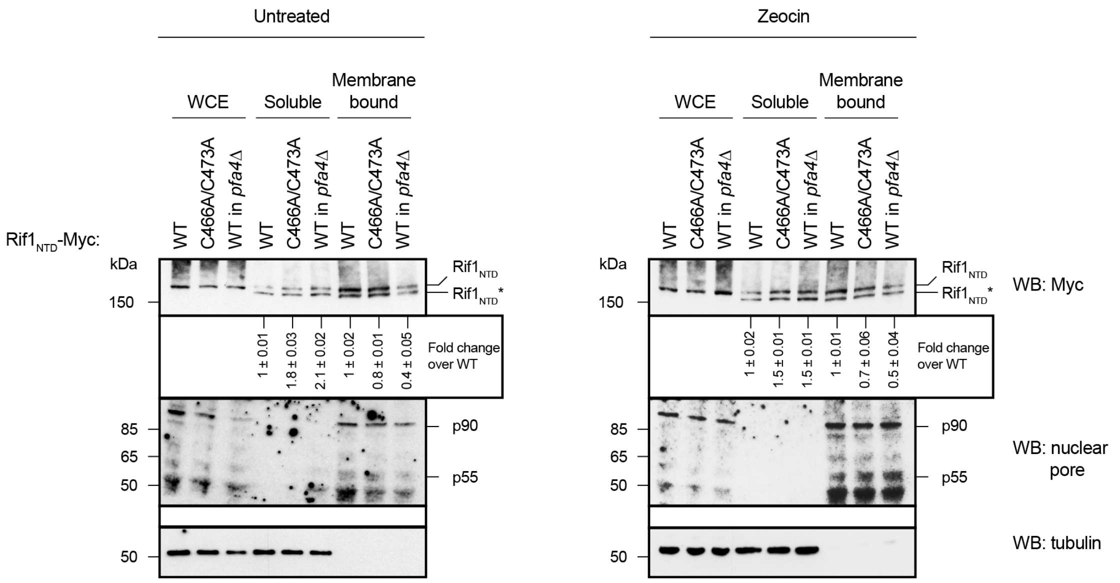

Supplementary Figure 5: Separation of Rif1$_{NTD}$ soluble and membrane-bound pools in untreated or DNA-damaging conditions. Untreated or Zeocin-treated cells expressing Myc-tagged Rif1$_{NTD}$ in the indicated backgrounds were spheroblasted, lysed, and subjected to differential centrifugation, separating soluble and membrane-bound fractions. Efficiency of fractionation was tested by Western blotting for tubulin and nuclear pore components, revealing the presence of nuclear membranes in the membrane-bound fractions. Western blots were quantified and signals for Rif1$_{NTD}$ from the indicated mutants are shown relative to wild-type. The data is presented as mean values ± s.e.m. (n = 3 independent experiments). WCE, whole cell extract. Rif1$_{NTD}$* denotes a likely N-terminally truncated form of Rif1$_{NTD}$.

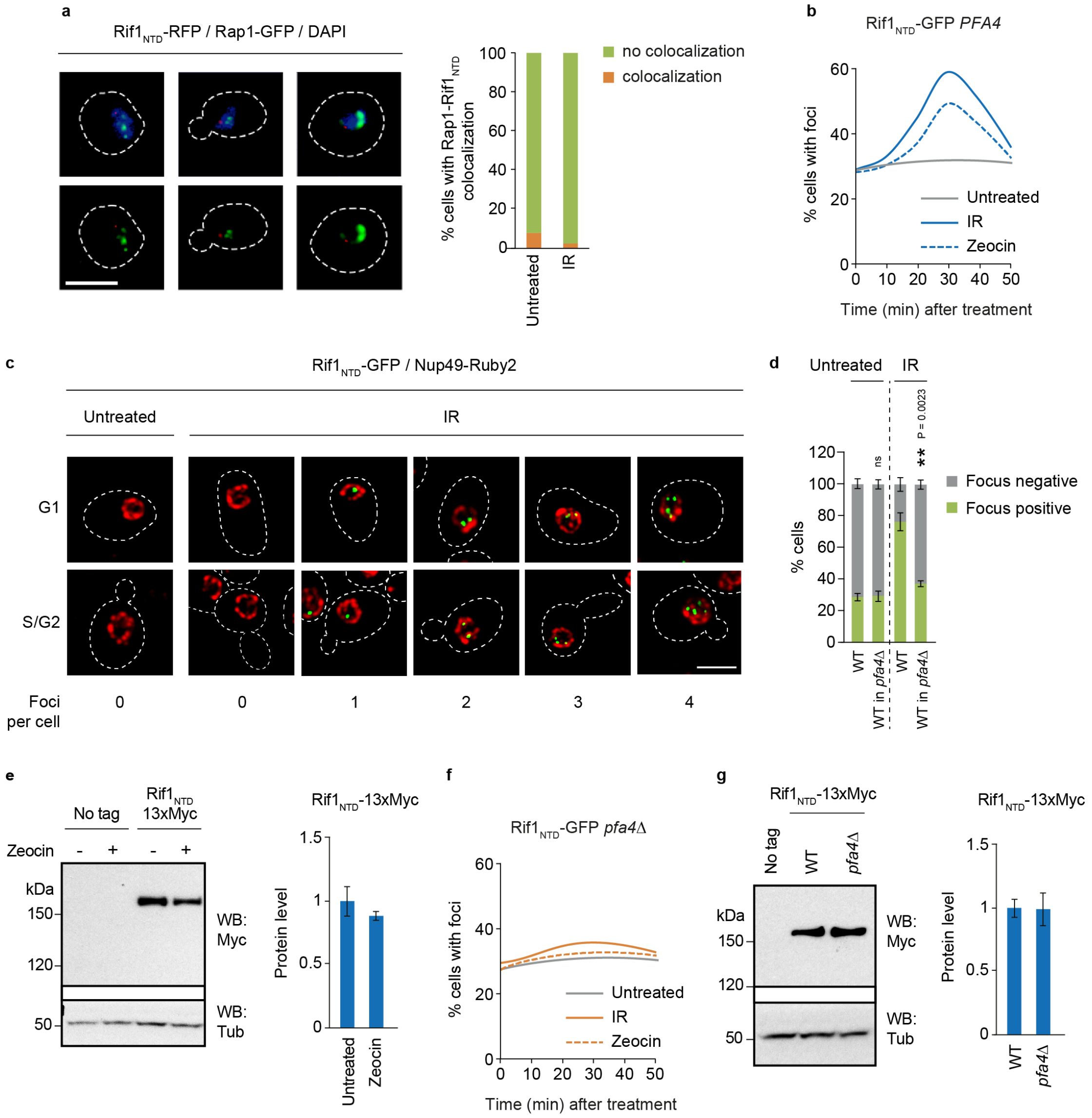

Supplementary Figure 6: In response to DNA damage, Pfa4 mediates a focal accumulation of Rif1 distinct from telomere clusters. (**a**) Confocal microscopy of cells expressing $Rif1_{NTD}$-RFP and Rap1-GFP as a marker of telomere clusters. Z-projected images show cells in G1 or S/G2 phase of the cell cycle with nuclear DNA stained by DAPI. Scale bar: 5 µm. Quantitation shows that $Rif1_{NTD}$ does not co-localize with Rap1 in untreated or IR-treated cells (n = 150 cells scored for each strain and condition, from 3 independent experiments). (**b**) Kinetics of $Rif1_{NTD}$ focus-formation in cells treated or not with IR or Zeocin, determined by confocal microscopy (n = 100 cells scored for each time-point, strain, and condition, from 3 independent experiments). (**c**) Confocal microscopy of cells expressing $Rif1_{NTD}$-GFP and Nup49-Ruby2, untreated or treated with IR (100 Gy). Z-projected images of cells in G1 or S/G2 phase of the cell cycle, illustrating the observed classes of focus-negative and $Rif1_{NTD}$ focus-positive (1-4 foci) cells. For untreated cells, only the main class (focus-negative, representing ~75% of cells within the population) is shown. Scale bar: 5 µm. (**d**) Quantification of focus-positive and

focus-negative cells for the indicated strains expressing wild-type or mutant $Rif1_{NTD}$-GFP, treated or not with IR. Results of 3 independent experiments (n ≥ 100 cells per experiment) are presented as mean values ± s.e.m. Statistical analysis by unpaired t-test, comparing wild-type to *pfa4*Δ in untreated and IR-treated conditions. (**e**) Western blot analysis and quantitation of Myc-tagged $Rif1_{NTD}$ in lysates of cells treated or not with Zeocin. $Rif1_{NTD}$ levels relative to wild-type are presented as mean values ± s.e.m. (n = 3 independent experiments). (**f**) Kinetics of $Rif1_{NTD}$ focus-formation in absence of Pfa4, determined as described for panel **b**. (**g**) Western blot analysis and quantitation of Myc-tagged $Rif1_{NTD}$ in lysates of *PFA4* and *pfa4*Δ cells. $Rif1_{NTD}$ levels relative to wild-type are presented as mean values ± s.e.m. (n = 3 independent experiments).

Supplementary Tables

Supplementary Table 1: Peptide transitions of selected Rif1$_{NTD}$ tryptic fragments

m/z	z	t start (min)	t stop (min)	Peptide sequence	Maximum injection time (ms)
725.4	2	18	24	L$_{484}$PLNSYDSANLDK$_{496}$	20
789.9	2	24	30	N$_{807}$DSSLVNFNIQISK$_{820}$	20
809.4	2	27	32	D$_{760}$QTHLESFSSLILK$_{773}$	20
889.9	2	32	38	I$_{1140}$ENGDDDYILELLEK$_{1154}$	20
979.5	2	35	45	T$_{954}$SLPGNPELFSGLLPFLR$_{971}$	20
1041.5	2	20	32	L$_{463}$YQ<u>C</u>IMLSPV<u>C</u>ETIPEK$_{479}$ with both cysteines CAM-labeled	500
1075.1	2	28	34	L$_{463}$YQ<u>C</u>IMLSPV<u>C</u>ETIPEK$_{479}$ with one CAM-labeled and one NEM-labeled cysteine	500
1109.1	2	30	40	L$_{463}$YQ<u>C</u>IMLSPV<u>C</u>ETIPEK$_{479}$ with both cysteines NEM-labeled	200

Underlined cysteine residues correspond to Rif1 C466 and C473.

Supplementary Table 2: *Saccharomyces cerevisiae* strains used in this study

**Strain name**	**Relevant genotype**	**Source**
BY4741	*MATa his3Δ1 leu2Δ0 met15Δ0 ura3Δ0*	Ref. 1
YUR-GF342	BY4741, *rif1::HIS3MX6*	Ref. 2
YUR-GF207	BY4741, *pfa4::KANMX6*	This study
YUR-GF300	BY4741, *pfa4::KANMX6 rif1::URA3*	This study
YUR-GF782	BY4741, *rif1-C466A*	This study
YUR-GF786	BY4741, *rif1-C473A*	This study
YUR-GF790	BY4741, *rif1-C466A-C473A*	This study
YUR-GF552	BY4741, *rif1*-K437E-K563E-K570E	Ref. 2
YUR-GF909	BY4741, *akr1::KANMX6*	YSC6273-201934477, ydr264c (Dharmacon)
YUR-GF910	BY4741, *akr2::KANMX6*	YSC6273-201923127, yor034c (Dharmacon)
YUR-GF911	BY4741, *erf2::KANMX6*	YSC6273-201937707, ylr246w (Dharmacon)
YUR-GF912	BY4741, *pfa3::KANMX6*	YSC6273-201918165, ynl326c (Dharmacon)
YUR-GF913	BY4741, *pfa4::KANMX6*	YSC6273-201922439, yol003c (Dharmacon)
YUR-GF914	BY4741, *pfa5::KANMX6*	YSC6273-201936007, ydr459c (Dharmacon)
YUR-GF915	BY4741, *swf1::KANMX6*	YSC6273-201935815, ydr126w (Dharmacon)
YUR-GF1028	BY4741, *pfa4-GFP::NATMX4*	This study
YUR-GF1029	UR-GF1028, *KANMX6*::*GAL*::*nup53*	This study
YUR-GF1050	YUR-GF1029, *HIS3MX6*::*GAL*::*pfa4*-GFP	This study
YUR-GF1048	BY4741, *HIS3MX6*::*GAL*::*nup53*	This study
YUR-GF1052	UR-GF1048, *sec61-GFP::KANMX6*	This study
YUR-GF1054	UR-GF1048, *hrd1-GFP::KANMX6*	This study
YUR-GF807	BY4741, *rif1-13MYC::KANMX6*	This study
YUR-GF1016	YUR-GF807, *rif1-C71A-13MYC::KANMX6*	This study

YUR-GF1019	YUR-GF807, *rif1-C466A-13MYC::KANMX6*	This study
YUR-GF809	YUR-GF807, *rif1-C473A-13MYC::KANMX6*	This study
YUR-GF810	YUR-GF807, *rif1-C466A-C473A-13MYC::KANMX6*	This study
YUR-GF1023	YUR-GF807, *rif1-C71A-C466A-C473A-13MYC::KANMX6*	This study
YUR-GF816	YUR-GF807, *pfa4::NATMX4*	This study
YUR-GF811	BY4741, *rif1Δ1323-13MYC::KANMX6*	This study
YUR-GF812	YUR-GF811, *rif1-C466A*	This study
YUR-GF814	YUR-GF811, *rif1-C473A*	This study
YUR-GF815	YUR-GF811, *rif1-C466A/C473A*	This study
YUR-GF1090	YUR-GF815, *rif1-K437E-C466A-C473A-K563E-K570E*	This study
YUR-GF819	YUR-GF811, *pfa4::NATMX4*	This study
YUR-GF861	BY4741, *HIS3MX6::GAL::rif1Δ1323-13MYC::KANMX6*	This study
YUR-GF870	YUR-GF861, *rif1-C466A-C473A*	This study
YUR-GF873	YUR-GF861, *pfa4::NATMX4*	This study
YUR-GF650	BY4741, *rif1Δ1323-GFP::NATMX4*	This study
YUR-GF739	YUR-GF650 *NUP49-RUBY2::KANMX6*	This study
YUR-GF853	YUR-GF739, *pfa4::HIS3MX6*	This study
YUR-GF1065	YUR-739, *rif1-C466A-C473A*	This study
YUR-GF1061	YUR-739, *rif1-K437E-K563E-K570E*	This study
YUR-GF1073	YUR-739, *rif1-K437E-C466A-C473A-K563E-K570E*	This study
GA-1168	ade2-1 his3-11,15 leu2-1,112 lys2 trp1-1 ura3 *RAD5+*, *rap1::RAP1-GFP/LEU2*	Ref. 3
YUR-GF167	GA-1168, *rif1Δ1323-RFP::HIS3MX6*	This study
JKM179	*MATα, Δho hml::ADE1 hmr::ADE1 ade3::GALHO ade1-100 leu2-3, 112 lys5 trp1::hisG ura3-52*	Ref. 4
GA-6844	JKM179, *CFP-NUP49 GFP-LacI:Leu2 MAT::LacO repeats:TRP1*	Ref. 5
YUR-GF445	GA-6844, *rif1::URA3*	Ref. 2
YUR-GF463	GA-6844, *pfa4::KANMX6*	This study
YUR-GF777	GA-6844, *pfa4::KANMX6 rif1::HIS3MX6*	This study
YUR-GF473	GA-6844, *yku70::KANMX6*	Ref. 2
YUR-GF889	GA-6844, *rif1-C71A*	This study

YUR-GF893	GA-6844, *rif1-C466A*	This study
YUR-GF932	GA-6844, *rif1-C473A*	This study
YUR-GF850	GA-6844, *rif1-C466A-C473A*	This study
YUR-GF948	GA-6844, *rif1-C71A-C466A-C473A*	This study
YUR-GF877	GA-6844, *rif1-C906A*	This study
YUR-GF881	GA-6844, *rif1-C1022A*	This study
YUR-GF916	GA-6844, *rif1-C1089A*	This study
YUR-GF918	GA-6844, *rif1-C1292A*	This study
YUR-GF885	GA-6844, *rif1-C906A-C1022A-C1089A-C1292A*	This study
YUR-GF1016	GA-6844, *rif1Δ1323-13MYC::KANMX6*	This study
YUR-GF1019	GF1016, *rif1-C466A*	This study
YUR-GF1020	GF1016, *rif1-C473A*	This study
YUR-GF1021	GF1016, *rif1-C466A-C473A*	This study
YUR-GF1022	GF1016, *rif1*-K437E-K563E-K570E	This study
YUR-GF1023	GF1016, *rif1*-K437E-C466A-C473A-K563E-K570E	This study
YUR-GF1024	GF1016, *pfa4::NATMX4*	This study
YMS331	W303-1A, *rif1::NatMX4*	Ref. 6
YKB2	*W303-1A, cdc7-4*	Ref. 7
YSM283	YKB2, *rif1Δ1323-13MYC::HIS3MX6*	This study
YSM291-1	YKB2, *cdc7-4 rif1::NatMX4*	This study
YSM636-2	YSM283, *rif1-C466A*	This study
YSM637	YSM283, *rif1-C473A*	This study
YSM638	YSM283, *rif1-C466A-C473A*	This study

Supplementary Table 3: Oligonucleotides used in this study

Name	DNA sequence (5′- 3′)	Application
EMSA1	TAGCAAGGCACTGGTAGAATTCGGCAGCGT	EMSA
EMSA2	ACGCTGCCGAATTCTACCAGTGCCTTGCTA	EMSA
GF288 FW	AAGAAGTCAACAGAAGGCA	*RIF1* sequencing
GF289 REV	GCCATTTTGATCTATTCTAC	*RIF1* sequencing
GF344 FW	ATGTCGAAAGATTTTTCAGATAAAAAG	*RIF1* sequencing
GF345 FW	GCAAACCAGGAAAATCAATTTTG	*RIF1* sequencing
GF346 FW	CCATTTGTAATCAGACTATATGTTC	*RIF1* sequencing
GF347 FW	CAATAGTTGTTTTACTGGAACTACT	*RIF1* sequencing
GF348 FW	ACGCTTCCTTATGTGAATGATCCAAG	*RIF1* sequencing
GF349 FW	GTCACGCAAAACAACAAAGATACACC	*RIF1* sequencing
GF350 FW	CTACTTCATGTGTCTATGTGGTCGAAC	*RIF1* sequencing
GF351 FW	GAATTTTGCGGAAATATGACCAGCG	*RIF1* sequencing
GF352 FW	CCTGACACTAAGTCGATGTATATTCC	*RIF1* sequencing
GF353 FW	CAGTGATAATCCTCTAAAACGACCTTC	*RIF1* sequencing
GF354 FW	GCGTAGTGATGAAGATGAACATGG	*RIF1* sequencing
GF355 FW	GCCAAGCCCACACAGAAGCAG	*RIF1* sequencing
GF356 REV	TCTCCTTTAAGCGGTCCCTTC	*RIF1* sequencing
GF357 REV	CCTGTCTTTGAACATGTTCAACTTGCC	*RIF1* sequencing
GF358 REV	TCGGTTAGCATGTGATTCTTGCTG	*RIF1* sequencing
GF359 REV	GGCGGCACTTCTAGAAAGCGG	*RIF1* sequencing
GF360 REV	AGCTCTGCTACGTGGTAACAGCTTC	*RIF1* sequencing
GF361 REV	TCATCCAATTCTAGAAACTTTTC	*RIF1* sequencing
GF362 REV	TCTGCATTCTTTCCCCCAATTAAC	*RIF1* sequencing
GF363 REV	CCGGTCATTGAAAGCCACAAATC	*RIF1* sequencing
GF364 REV	CCATTATTTCAGTGAAAGTGCTAATG	*RIF1* sequencing
GF365 REV	ACACTTTCATTATCAACTAAACCATGG	*RIF1* sequencing
GF366 REV	CGCAATCACTTGAAGACGCTAG	*RIF1* sequencing

GF321 FW	AGGATTGCCATTGCAAAATCGTTTTTGTGGT CAATTTGCACGGATCCCCGGGTTAATTAA	*RIF1* gene deletion
GF322 REV	TTAATTTATTGCCATTTTGATCTATTCTACA TACTAACAAGCGCGTTGGCCGATTCATTA	*RIF1* gene deletion
GF384 FW	TCTCAAGATATCCAGGTTCCGGCTACGCAG GGAATGAAAGAGCCTGGTGACGGTGCTGGT TTA	*RIF1* C-terminal tagging (RFP)
GF385 REV	TGTAATTAATTTATTGCCATTTTGATCTATT CTACATACTAACAATCGATGAATTCGAGCT CG	*RIF1* C-terminal tagging (RFP)
GF430 FW	GTAATTAATTTATTGCCATTTTGATCTATTC TACATACTAACAAGAATTCGAGCTCGTTTA AAC	*RIF1* C-terminal tagging (GFP, 13xMyc)
GF432 REV	AGATATCCAGGTTCCGGCTACGCAGGGAAT GAAAGAGCCTCGGATCCCCGGGTTAATTAA	*RIF1* C-terminal tagging (GFP, 13xMyc)
GF1124 FW	GATCTATCGCCAACGCCAAAGAGGCGAAAG CTAGCGTCTTCAAGTGATGCTGAGAATAAA CAATTTGATTTATCAGCTATTAACAAGAATT TATATCC	*RIF1 C71A* mutation: recombination template
GF1125 REV	GGATATAAATTCTTGTTAATAGCTGATAAA TCAAATTGTTTATTCTCAGCATCACTTGAAG ACGCTAGCTTTCGCCTCTTTGGCGTTGGCGA TAGATC	*RIF1 C71A* mutation: recombination template
GF1126 FW	GCGATCTATCGCCAACGCCAAAGAGGCGAA AGCTAGCGTCTTCAAGTGATGAGCTCGTTTT CGACACTGG	*RIF1 C466A* mutation: CORE cassette amplification
GF1127 REV	TCTGGATATAAATTCTTGTTAATAGCTGATA AATCAAATTGTTTATTCTCTCCTTACCATTA AGTTGATC	*RIF1 C466A* mutation: CORE cassette amplification
GF949 FW	TACTAAAAAAATGCTTAGATTTTGTTGATG AACATGAAAGGATTTACCAGGCTATCATGC TATCTCCAGTATGCGAAACAATCCCGGAAA AATTTTTATCTAA	*RIF1 C466A* mutation: recombination template
GF950 REV	TTAGATAAAAATTTTTCCGGGATTGTTTCGC ATACTGGAGATAGCATGATAGCCTGGTAAA TCCTTTCATGTTCATCAACAAAATCTAAGCA TTTTTTTAGTA	*RIF1 C466A* mutation: recombination template
GF951 FW	TACTAAAAAAATGCTTAGATTTTGTTGATG AACATGAAAGGATTTACCAGGAGCTCGTTT TCGACACTGG	*RIF1 C466A* mutation: CORE cassette amplification
GF952 REV	TTAGATAAAAATTTTTCCGGGATTGTTTCGC ATACTGGAGATAGCATGATTCCTTACCATT AAGTTGATC	*RIF1 C466A* mutation: CORE cassette amplification
GF953 FW	TTGTTGATGAACATGAAAGGATTTACCAGT GTATCATGCTATCTCCAGTAGCCGAAACAA TCCCGGAAAAATTTTTATCTAAACTACCGTT AAATTCATATGA	*RIF1 C473A* mutation: recombination template

GF954 REV	TCATATGAATTTAACGGTAGTTTAGATAAA AATTTTTCCGGGATTGTTTCGGCTACTGGAG ATAGCATGATACACTGGTAAATCCTTTCAT GTTCATCAACAA	*RIF1 C473A* mutation: recombination template
GF955 FW	TTGTTGATGAACATGAAAGGATTTACCAGT GTATCATGCTATCTCCAGTAGAGCTCGTTTT CGACACTGG	*RIF1 C473A* mutation: CORE cassette amplification
GF956 REV	TCATATGAATTTAACGGTAGTTTAGATAAA AATTTTTCCGGGATTGTTTCTCCTTACCATT AAGTTGATC	*RIF1 C473A* mutation: CORE cassette amplification
GF961 FW	GCTTAGATTTTGTTGATGAACATGAAAGGA TTTACCAGGCTATCATGCTATCTCCAGTAGC CGAAACAATCCCGGAAAAATTTTTATCTAA ACTACCGTT	*RIF1 C466A/473A* mutations: recombination template
GF962 REV	AACGGTAGTTTAGATAAAAATTTTTCCGGG ATTGTTTCGGCTACTGGAGATAGCATGATA GCCTGGTAAATCCTTTCATGTTCATCAACAA AATCTAAGC	*RIF1 C466A/473A* mutations: recombination template
GF963 FW	TACTAAAAAAATGCTTAGATTTTGTTGATG AACATGAAAGGATTTACCAGGAGCTCGTTT TCGACACTGG	*RIF1 C466A/473A* mutations: CORE cassette amplification
GF964 REV	TCATATGAATTTAACGGTAGTTTAGATAAA AATTTTTCCGGGATTGTTTCTCCTTACCATT AAGTTGATC	*RIF1 C466A/473A* mutations: CORE cassette amplification
GF1132 FW	TGTGAACAAAGTTCCGAATGAAAATTCCAT TGAAAATTTCTTGGACCTAGCTTTGAAGCTC AGCTTTCCAGTTAATTTGTTTACACTACTTC ATGTGTC	*RIF1 C906A* mutation: recombination template
GF1133 REV	GACACATGAAGTAGTGTAAACAAATTAACT GGAAAGCTGAGCTTCAAAGCTAGGTCCAAG AAATTTTCAATGGAATTTTCATTCGGAACTT TGTTCACA	*RIF1 C906A* mutation: recombination template
GF1134 FW	TTGTGAACAAAGTTCCGAATGAAAATTCCA TTGAAAATTTCTTGGACCTAGAGCTCGTTTT CGACACTGG	*RIF1 C906A* mutation: CORE cassette amplification
GF1135 REV	ATAGACACATGAAGTAGTGTAAACAAATTA ACTGGAAAGCTGAGCTTCAATCCTTACCAT TAAGTTGATC	*RIF1 C906A* mutation: CORE cassette amplification
GF1136 FW	ACCACGTAGCAGAGCTTCATATTTTGCAGC AAACATAAAACTATTCAAGGCTTCCGAACA GCTTACATTAGTTCGTTGGCTGTTGAAGGGT CAACAACT	*RIF1 C1022A* mutation: recombination template

GF1137 REV	AGTTGTTGACCCTTCAACAGCCAACGAACT AATGTAAGCTGTTCGGAAGCCTTGAATAGT TTTATGTTTGCTGCAAAATATGAAGCTCTGC TACGTGGT	*RIF1 C1022A* mutation: recombination template
GF1138 FW	TACCACGTAGCAGAGCTTCATATTTTGCAG CAAACATAAAACTATTCAAGGAGCTCGTTT TCGACACTGG	*RIF1 C1022A* mutation: CORE cassette amplification
GF1139 REV	TCAAGTTGTTGACCCTTCAACAGCCAACGA ACTAATGTAAGCTGTTCGGATCCTTACCATT AAGTTGATC	*RIF1 C1022A* mutation: CORE cassette amplification
GF1140 FW	GGCGATGGCAAATCCCATTGAACCATTATT CAGTGGCCTATTGAATTTTGCTATAAAGAA TAATATGGCGGATCATTTGGATGAATTTTGC GGAAATAT	*RIF1 C1089A* mutation: recombination template
GF1141 REV	ATATTTCCGCAAAATTCATCCAAATGATCC GCCATATTATTCTTTATAGCAAAATTCAATA GGCCACTGAATAATGGTTCAATGGGATTTG CCATCGCC	*RIF1 C1089A* mutation: recombination template
GF1142 FW	TGGCGATGGCAAATCCCATTGAACCATTAT TCAGTGGCCTATTGAATTTTGAGCTCGTTTT CGACACTGG	*RIF1 C1089A* mutation: CORE cassette amplification
GF1143 REV	GTCATATTTCCGCAAAATTCATCCAAATGAT CCGCCATATTATTCTTTATTCCTTACCATTA AGTTGATC	*RIF1 C1089A* mutation: CORE cassette amplification
GF1144 FW	AAGTAAGGAAATTGAAGCTATACCTGACAC TAAGTCGATGTATATTCCAGCTGAAGGGAG TGAAAATAAACTTTCAAACTTACAGAGAAA AGTGGATTC	*RIF1 C1292A* mutation: recombination template
GF1145 REV	GAATCCACTTTTCTCTGTAAGTTTGAAAGTT TATTTTCACTCCCTTCAGCTGGAATATACAT CGACTTAGTGTCAGGTATAGCTTCAATTTCC TTACTT	*RIF1 C1292A* mutation: recombination template
GF1146 FW	CAAGTAAGGAAATTGAAGCTATACCTGACA CTAAGTCGATGTATATTCCAGAGCTCGTTTT CGACACTGG	*RIF1 C1292A* mutation: CORE cassette amplification
GF1147 REV	TGAGAATCCACTTTTCTCTGTAAGTTTGAAA GTTTATTTTCACTCCCTTCTCCTTACCATTAA GTTGATC	*RIF1 C1292A* mutation: CORE cassette amplification
GF1169 FW	ACGTTATTACAGGATTGCCATTGCAAAATC GTTTTTGTGGTCAATTTGCAGAATTCGAGCT CGTTTAAAC	*GAL1* promoter exchange for *RIF1*

GF1170 REV	TCAATTCGATCTATCGTATGTTTCTTTTTATC TGAAAAATCTTTCGACATCATTTGAGATCC GGGTTTT	*GAL1* promoter exchange for *RIF1*
GF1432 FW	CATGAAAGGATTTACCAGGCTATCATGCTA TCTCCAGTAGCCGAAACAATCCC	*RIF1 C466A/C473A* site-directed mutagenesis in pFastBac vector
GF1433 REV	GGGATTGTTTCGGCTACTGGAGATAGCATG ATAGCCTGGTAAATCCTTTCATG	*RIF1 C466A/C473A* site-directed mutagenesis in pFastBac vector
GF1707 FW	TCCAGTATGCGAAACAATCCGTTTT	*RIF1 C466A/473A* mutations: gRNA
GF1708 REV	GGATTGTTTCGCATACTGGAGATCA	*RIF1 C466A/473A* mutations: gRNA
GF1709 FW	TTAGATTTTGTTGATGAACATGAAAGAATTT ACCAGGCTATCATGCTATCTCCAGTAGCTG AAACAATCCCAGAAAAATTTTTATCTAAAC TACCGTT	*RIF1 C466A/473A* mutations: recombination template
GF1710 REV	AACGGTAGTTTAGATAAAAATTTTTCTGGG ATTGTTTCAGCTACTGGAGATAGCATGATA GCCTGGTAAATTCTTTCATGTTCATCAACAA AATCTAA	*RIF1 C466A/473A* mutations: recombination template
GF325 FW	CTACCTCTCTTACTACGCGCTTATG	*PFA4* sequencing
GF326 REV	ACACGTCATTAGCATAGTATGTAATC	*PFA4* sequencing
GF1280 REV	CGCCAGATATCAGGGGGTGG	*PFA4* sequencing
GF1284 FW	GGCCATATGGTGTTCCTACG	*PFA4* sequencing
GF1382 FW	GGCTCTCCTATTACTTGGCCATCTG	*PFA4* sequencing
GF1383 REV	GAGCTGCCATGATTGAAGACGGTG	*PFA4* sequencing
GF1384 REV	CTAGCCGATCCATATCCCACGACTC	*PFA4* sequencing
GF319 FW	GTATAACATGAATTTTAAAAACTGAGTTGA TCGTTAAACCCGGATCCCCGGGTTAATTAA	*PFA4* gene deletion
GF320 REV	TTATACATACATATACTTGATATTCCCATGA ATGAGTATTGCGCGTTGGCCGATTCATTA	*PFA4* gene deletion
GF1281 FW	ACGACTGGGGTGAATCACTAGACGATTTTG GAGTGGATGTTGATATGGAACGGATCCCCG GGTTAATTAA	*PFA4* C-terminal tagging (GFP)
GF1282 REV	AAGGGACAGTTTATACATACATATACTTGA TATTCCCATGAATGAGTATTGAATTCGAGCT CGTTTAAAC	*PFA4* C-terminal tagging (GFP)

GF1274 FW	GTGGTGATGGTGTATAACATGAATTTTAAA AACTGAGTTGATCGTTAAACCGAATTCGAG CTCGTTTAAAC	*GAL1* promoter exchange for *PFA4*
GF1275 REV	AACGTTGGTATAGCAATTCCTAACCAAGGC CACCTTAACTTTACTGGCATCATTTTGAGAT CCGGGTTTT	*GAL1* promoter exchange for *PFA4*
GF592 FW	CGAAATCTTGAGATCGGGCGTTCG	*YKU70* sequencing
GF593 REV	GGTGAGCCTGAAATTTCAAATTGGGC	*YKU70* sequencing
GF590 FW	TGTTAAGTGACTCTAAGCCTGATTTTAAAAC GGGAATATTCGGATCCCCGGGTTAATTAA	*YKU70* gene deletion
GF591 REV	TTGTATGTAACGTTATAGATATGAAGGATTT CAATCGTCTGCGCGTTGGCCGATTCATTA	*YKU70* gene deletion
GF889 FW	GGCGCCCCAAATAACCCTAATTC	*NUP49* sequencing
GF890 REV	CCTCTAAGACGCCATTATGGTGACTG	*NUP49* sequencing
GF891 FW	CGGTTCGCTTGTGCGAACGC	*NUP49* sequencing
GF1167 FW	GTTTATGGATATCGCTGAGAGAATCGCCGT GTTACATCAAAAAACGAAAACACTGGCATC ATTGAGCATAGGTGACGGTGCTGGTTTA	*NUP49* C-terminal tagging (Ruby2)
GF1168 REV	GTATAAATTACATTTGTACAAGACATTTGTA CTTGTTATACGCACTATATAAACTTTCAGGG CGATTTACTCGATGAATTCGAGCTCG	*NUP49* C-terminal tagging (Ruby2)
GF1687 FW	CGGTGCTACCATCGGTGCTC	*SEC61* sequencing
GF1688 REV	GCGTATGATATTGTTAATCGGCGGTTA	*SEC61* sequencing
GF1685 FW	GGAAGGTGGGTTTACTAAGAACCTCGTTCC AGGATTTTCTGATTTGATGGGTGACGGTGCT GGTTTA	*SEC61* C-terminal tagging (GFP)
GF1686 REV	GTGGCTAAATGCGATTTTTTTTTCTTTGGA TATTATTTTCATTTTATATTCGATGAATTCG AGCTCG	*SEC61* C-terminal tagging (GFP)
GF1691 FW	CTGTTGGGTCAAGCCGATCAGC	*HRD1* sequencing
GF1692 REV	GCTGATCGATGTAGCATGTGTACGC	*HRD1* sequencing
GF1689 FW	CGAGCAAATTGCCAAGAAAATTGTCATACC AGATAAATTTATCCAGCATATCGGTGACGG TGCTGGTTTA	*HRD1* C-terminal tagging (GFP)
GF1690 REV	CCAGTAGTTTTTTTCTTTAAAAAAAACTATG TATAATATAAAACATGCAATTCGATGAATT CGAGCTCG	*HRD1* C-terminal tagging (GFP)
GF1704 FW	GATGGGCATTACCCGACCGAC	*NUP53* sequencing
GF1705 REV	GTTCATGTTGTCCCTGTGATTCGGG	*NUP53* sequencing
GF1702 FW	CTCAGCTAGCCCAAATTCTCTCTGCACTCTC AATAACATAGCTTTTTAAAGAATTCGAGCT CGTTTAAAC	*GAL1* promoter exchange for *NUP53*
GF1703 REV	GAAACATTGGTGAACCTGCTACTATTCTCTT GTTTTTGAAGGTCTGCCATCATTTTGAGATC CGGGTTTT	*GAL1* promoter exchange for *NUP53*

SG2285 (ref. 8)	AATATGGGACTACTTCGCGCAACA	*MAT*α qPCR for HO-cut efficiency
SG2286 (ref. 8)	CGTCACCACGTACTTCAGCATAA	*MAT*α qPCR for HO-cut efficiency
SG8440 (ref. 8)	CTCTCCCTTGGTGTTTCCAA	*MAT*α qPCR: 0.7 kb from HO-cut site
SG8441 (ref. 8)	GAAAAGATTGGCCGTCAAAA	*MAT*α qPCR: 0.7 kb from HO-cut site
SG8459 (ref. 8)	TGCGATGAAGTCAACGAATTA	*MAT*α qPCR: 2.5 kb from HO-cut site
SG8460 (ref. 8)	GAGCACTTTTACCGGCAGTT	*MAT*α qPCR: 2.5 kb from HO-cut site
SG8448 (ref. 8)	CAATGCCTTCCTTCTCCAAA	*MAT*α qPCR: 4.2 kb from HO-cut site
SG8449 (ref. 8)	ACCTGAGCGACGAGAAATTG	*MAT*α qPCR: 4.2 kb from HO-cut site
SG525 (ref. 8)	AATTGGATTTGGCTAAGCGTAATC	*SMC2* qPCR
SG526 (ref. 8)	CTCCAATGTCCCTCAAAATTTCTT	*SMC2* qPCR
SM421 FW	AACGTCTAGCTGAGCATGTGAG	*MAT*α ChIP qPCR: 0.3 kb from HO-cut site
SM422 REV	CGAGAGGAAGGAACAGGAATCTG	*MAT*α ChIP qPCR: 0.3 kb from HO-cut site
SM425 FW	GCGATGAAGTCAACGAATTATTCGG	*MAT*α ChIP qPCR: 2.5 kb from HO-cut site
SM426 REV	CGGTATGTCTCGAGTATTACC	*MAT*α ChIP qPCR: 2.5 kb from HO-cut site
SM419 FW	TCGATGAAGATGGTTCTCCTGC	*MAT*α ChIP qPCR: 4 kb from HO-cut site
SM420 REV	ACTCTCAGACTCCAGGTCCAATC	*MAT*α ChIP qPCR: 4 kb from HO-cut site
SM ACT1 FW	AAGCCGGTTTTGCCGG	*ACT1* qPCR
SM ACT1 REV	TTGTGTCTTGGTCTACCGACG	*ACT1* qPCR

Supplementary References

1. Brachmann, C.B. et al. Designer deletion strains derived from *Saccharomyces cerevisiae* S288C: a useful set of strains and plasmids for PCR-mediated gene disruption and other applications. *Yeast* **14**, 115-32 (1998).
2. Mattarocci, S. et al. Rif1 maintains telomeres and mediates DNA repair by encasing DNA ends. *Nat. Struct. Mol. Biol.* **24**, 588-595 (2017).
3. Schober, H. et al. Controlled exchange of chromosomal arms reveals principles driving telomere interactions in yeast. *Genome Res.* **18**, 261-71 (2008).
4 Lee, S.E., Paques, F., Sylvan, J. & Haber, J.E. Role of yeast SIR genes and mating type in directing DNA double-strand breaks to homologous and non-homologous repair paths. *Curr. Biol.* **9**, 767-70 (1999).
5. Horigome, C. et al. PolySUMOylation by Siz2 and Mms21 triggers relocation of DNA breaks to nuclear pores through the Slx5/Slx8 STUbL. *Genes Dev.* **30**, 931-45 (2016).
6. Mattarocci, S. et al. Rif1 controls DNA replication timing in yeast through the PP1 phosphatase Glc7. *Cell Rep.* **7**, 62-9 (2014).
7. Bousset, K. & Diffley, J.F. The Cdc7 protein kinase is required for origin firing during S phase. *Genes Dev.* **12**, 480-90 (1998).
8. Marcomini, I. et al. Asymmetric processing of DNA ends at a double-strand break leads to unconstrained dynamics and ectopic translocation. *Cell Rep.* **24**, 2614–2628 (2018).
