# Supplementary material for: Rif1 S-acylation mediates DNA double-strand break repair at the inner nuclear membrane
Source: Nat Commun. 2019 Jun 10;10:2535. doi: 10.1038/s41467-019-10349-z (PMC6557901; doi:10.1038/s41467-019-10349-z)
Supplement: Supplementary file 3 — Reporting Summary [file 41467_2019_10349_MOESM3_ESM.pdf]

nature research

Corresponding author(s): Urlich Rass

Reporting Summary

Nature Research wishes to improve the reproducibility of the work that we publish. This form provides structure for consistency and transparency in reporting. For further information on Nature Research policies, see Authors & Referees and the Editorial Policy Checklist.

Statistical parameters

When statistical analyses are reported, confirm that the following items are present in the relevant location (e.g. figure legend, table legend, main text, or Methods section).

n/a	Confirmed	
☐	☒	The exact sample size (n) for each experimental group/condition, given as a discrete number and unit of measurement
☐	☒	An indication of whether measurements were taken from distinct samples or whether the same sample was measured repeatedly
☐	☒	The statistical test(s) used AND whether they are one- or two-sided<br>*Only common tests should be described solely by name; describe more complex techniques in the Methods section.*
☒	☐	A description of all covariates tested
☐	☒	A description of any assumptions or corrections, such as tests of normality and adjustment for multiple comparisons
☐	☒	A full description of the statistics including central tendency (e.g. means) or other basic estimates (e.g. regression coefficient) AND variation (e.g. standard deviation) or associated estimates of uncertainty (e.g. confidence intervals)
☐	☒	For null hypothesis testing, the test statistic (e.g. F, t, r) with confidence intervals, effect sizes, degrees of freedom and P value noted<br>*Give P values as exact values whenever suitable.*
☒	☐	For Bayesian analysis, information on the choice of priors and Markov chain Monte Carlo settings
☒	☐	For hierarchical and complex designs, identification of the appropriate level for tests and full reporting of outcomes
☒	☐	Estimates of effect sizes (e.g. Cohen's d, Pearson's r), indicating how they were calculated
☐	☒	Clearly defined error bars<br>*State explicitly what error bars represent (e.g. SD, SE, CI)*

Our web collection on statistics for biologists may be useful.

Software and code

Policy information about availability of computer code

Data collection	Full detail of softwares used for data collection (including producer and version) can be found in the Methods section.
Data analysis	Full detail of softwares used for data analysis (including producer and version) can be found in the Methods section.

For manuscripts utilizing custom algorithms or software that are central to the research but not yet described in published literature, software must be made available to editors/reviewers upon request. We strongly encourage code deposition in a community repository (e.g. GitHub). See the Nature Research guidelines for submitting code & software for further information.

Data

Policy information about availability of data

All manuscripts must include a data availability statement. This statement should provide the following information, where applicable:

- Accession codes, unique identifiers, or web links for publicly available datasets
- A list of figures that have associated raw data
- A description of any restrictions on data availability

The data underlying Figures Fig. 1b-d, 2c, 3a, b, d, 4d, e, 6b, c is provided as Source Data file. Other data supporting the findings of this study are available from the corresponding author upon reasonable request. Structural figures are based on published structures (PDB: 5NVR and PDB: 5NW5). The mass spectrometry

proteomics data have been deposited at ProteomeXchange via PRIDE and can be accessed using access code PXD012137 [http://proteomecentral.proteomexchange.org/cgi/GetDataset?ID=PXD012137].

Field-specific reporting

Please select the best fit for your research. If you are not sure, read the appropriate sections before making your selection.

☒ Life sciences ☐ Behavioural & social sciences ☐ Ecological, evolutionary & environmental sciences

For a reference copy of the document with all sections, see nature.com/authors/policies/ReportingSummary-flat.pdf

Life sciences study design

All studies must disclose on these points even when the disclosure is negative.

Sample size	Sample size was chosen to ensure an adequate statistical power.
Data exclusions	No data were excluded from analysis.
Replication	Reproducibility of experimental findings was assessed by performing experiments with independent biological replicates separately collected. Experimental variation is reported in the applicable figures as standard error of the mean.
Randomization	Not relevant
Blinding	Data underlying Figures 1b-d, 2c, 3a, 6b,c,d and Supplementary Figures 1b, 2a, 3a, 6a,b,c,d, f were collected with blinding.

Reporting for specific materials, systems and methods

Materials & experimental systems

n/a	Involved in the study	
☐	☒	Unique biological materials
☐	☒	Antibodies
☒	☐	Eukaryotic cell lines
☒	☐	Palaeontology
☒	☐	Animals and other organisms
☒	☐	Human research participants

Methods

n/a	Involved in the study	
☒	☐	ChIP-seq
☒	☐	Flow cytometry
☒	☐	MRI-based neuroimaging

Unique biological materials

Policy information about availability of materials

Obtaining unique materials	Full details of commercial reagents used in this study, including producer and catalog number, are reported in the Methods section. Primer sequences are presented in Supplementary Table 3. Commercial yeast strains used for this study are reported in in Supplementary Table 2 with producer and catalog numbers. Non-commercial yeast strains generated for this study are reported in Supplementary Table 2 and are available from the corresponding author upon reasonable request.

Antibodies

Antibodies used	Full detail of antibodies used for Western Blotting and ChIP analyses (including dilution used, producer, catalog number and RRID) can be found in the Methods section.
Validation	The antibodies used in this study are widely used and validation as well as relevant literature references can be found on the manufacturers' web sites using the provided RRIDs.
